# Design, Implementation and Validation of a GNSS Measurement Exclusion and Weighting Function with a Dual Polarized Antenna †

**DOI:** 10.3390/s18124483

**Published:** 2018-12-18

**Authors:** Daniel Egea-Roca, Antonio Tripiana-Caballero, José A. López-Salcedo, Gonzalo Seco-Granados, Wim De Wilde, Bruno Bougard, Jean-Marie Sleewaegen, Alexander Popugaev

**Affiliations:** 1 Department of Telecommunications and Systems Engineering, IEEC-CERES, Universitat Autònoma de Barcelona (UAB), Barcelona 08193, Spain; antonio.tripiana@uab.cat (A.T.-C.); Jose.Salcedo@uab.cat (J.A.L.-S.); Gonzalo.Seco@uab.cat (G.S.-G.); 2 Septentrio, Leuven 3001, Belgium; wim.dewilde@septentrio.com (W.D.W.); bruno.bougard@septentrio.com (B.B.); jm.sleewaegen@septentrio.com (J.-M.S.); 3 Fraunhofer IIS, 91058 Erlangen, Germany; alexander.popugaev@iis.fraunhofer.de

**Keywords:** polarization, multipath mitigation, range error, modeling

## Abstract

Global Navigation Satellite Systems (GNSSs) have become a ubiquitous tool for our modern society to carry out vital tasks such as transportation, civil engineering or precision agriculture. This breath has reached the realm of safety-critical applications such as time management of critical infrastructures or autonomous vehicles, in which GNSS is an essential tool nowadays. Unfortunately, current GNSS performance is not enough to fulfill the requirements of these professional and critical applications. For this reason, the FANTASTIC project was launched to boost the adoption of these applications. The project was funded by the European GNSS agency (GSA) in order to enhance the robustness and accuracy of GNSS in harsh environments. This paper presents the part related to the development of a weighting and exclusion function with a dual circularly polarized antenna. The idea is to reduce the effects of multipath by weighting and/or excluding those measurements affected by multipath. The observables and other metrics obtained from a dual polarized antenna will be exploited to define an exclusion threshold and to provide the weights. Real-world experiments will show the improvement in the positioning solution, using all available constellations, obtained with the developed technique.

## 1. Introduction

Professional applications based on global navigation satellite systems (GNSSs) are getting a significant boost in terms of adoption and absolute performance, mainly driven by the growing number of satellites and signals. Furthermore, the wide use of correction services like SBAS (e.g., EGNOS), precise point positioning and regional and nation-wide real time kinematics (RTK) networks, can offer sub-decimeter accuracy. Nevertheless, this cutting edge accuracy is not enough for a large variety of emerging applications posing very stringent requirements. These applications include the control of driverless machineries in precision farming, autonomous vehicles, and GNSS-based systems resilient to interference, just to mention a few.

For the above reason, the FANTASTIC project was launched in order to develop enabling technologies that allow a leap of professional applications. One of the main tasks of the FANTASTIC project is to demonstrate the improvement of the RTK positioning in harsh environments, where the received signal is likely to be corrupted by obstacles (foliage, buildings, mountains, ...). To enhance robustness, the work focuses on a dual circularly polarized multi-band antenna, a new strategy to combine inertial measurement unit (IMU) and GNSS measurements and on interference mitigation algorithms. This paper will focus on the part corresponding to the antenna. It has two orthogonal polarization outputs (Right-Hand Circular Polarization (RHCP) and Left-Hand Circular Polarization (LHCP) components). Since GNSS satellites transmit RHCP signals, the underlying idea with this configuration is that multipath may affect both the RHCP and the LHCP components, while the direct or genuine signal appears only on the RHCP component. The aim of this paper is to show the developed function in the FANTASTIC project to weight and exclude observables based on the above concept. The goal of the proposed weighting and exclusion (WE) function is to reduce the effects of multipath into the Position, Velocity and Time (PVT) solution.

### 1.1. Related Work

In the last decade, many efforts have been made to reduce the effects of multipath in the PVT solution. Actually, there are many techniques to do so, ranging from the classical narrow or strobe correlators [1] to more sophisticated techniques such as the MEDLL [2]. These techniques are either not designed to mitigate carrier phase effects or are usually too complex to be implemented in mass-market receivers. Other schemes include site-dependent techniques trying to model the multipath propagation with in-site multipath calibrations [3] or with the use of external information such as cameras [4]. Unfortunately, these techniques are only suited to static receivers with very specific multipath propagation or they need additional hardware/components that are not usually available in traditional GNSS receivers.

Furthermore, none of the techniques listed above completely eliminate the effects of multipath or suit all GNSS applications. The most effective techniques are those based on antenna arrays [5], which can cover most multipath situations. Nevertheless, antenna arrays are often not suited in some kinematic applications due to the size of the array, which is usually bulky. In this regard, multipath mitigation techniques using dual polarization antennas may provide a good alternative due to the reduction of size, as they have the same size as a single antenna element. The first studies of dual polarization antennas for GNSS multipath mitigation can be found in Refs. [6,7,8], which consider in-lab experiments or simulation demonstrations. It was not until some years later, though, that real-world conditions were analyzed in Refs. [9,10,11]. These works demonstrated for the first time the capability of dual polarization antennas to detect Non-Line-of-Sight (NLOS) and then to improve the positioning accuracy under real working conditions.

### 1.2. Contributions

For the best of the authors’ knowledge, there is no literature available evaluating the case of RTK phase-based positioning, and for the application of a weighting function, there is only the work in Ref. [12]. Based on these observations, the contribution of this paper is twofold. On the one hand, we illustrate the design of an exclusion function with a dual polarized antenna and its effects on the performance of the RTK phase-based positioning in real-world conditions. On the other hand, we introduce a novel weighting function based on a dual polarized antenna. This was briefly introduced in Ref. [13]. In this work, though, we provide a more extensive and complete analysis of the developed algorithm. In particular, we give more details about the set-up and hardware used to capture the data within the framework of the FANTASTIC project. This includes the description and properties of the developed dual polarized antenna during the project. Furthermore, in this paper, we describe the procedure carried out to obtain the results presented in this work. Finally, it is worth mentioning that the real-world results presented in Ref. [13] were a summary of the whole set of PVT results obtained within the FANTASTIC project. In this work, we additionally show the fine-tuning of the proposed algorithm.

The rest of the paper is organized as follows: Section 2 introduces the set-up and scenarios considered for the measurement collection campaign. We also show some preliminary results before entering into details of the proposed WE function in Section 3. Finally, Section 4 shows the PVT results after applying the proposed WE function, while Section 5 concludes the paper.

## 2. Set-Up and Hardware Description

Multipath propagation is one of the main limiting factors on the accuracy of GNSS for professional applications operating in harsh environments like dense urban areas or foliage zones [14,15,16,17]. In particular, the case when the direct Line-of-Sight (LOS) signal is not present, known as NLOS signal reception, is very dangerous because these kind of signals can induce very large errors depending on the distance of the reflector. These effects can be sensed by using a dual-polarized antenna, as we show in this section by introducing the used hardware and the collection campaign carried out within the framework of the FANTASTIC project. This set-up is the cornerstone for the development of multipath mitigation techniques with dual-polarized antennas.

### 2.1. Dual-Polarized Antenna

**Figure 1:**
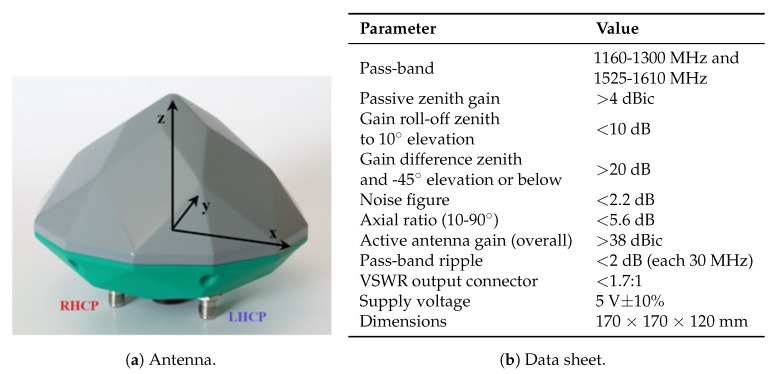
Developed 2pol antenna within the framework of the FANTASTIC project (**a**) and its main characteristics (**b**).

**Figure 2:**
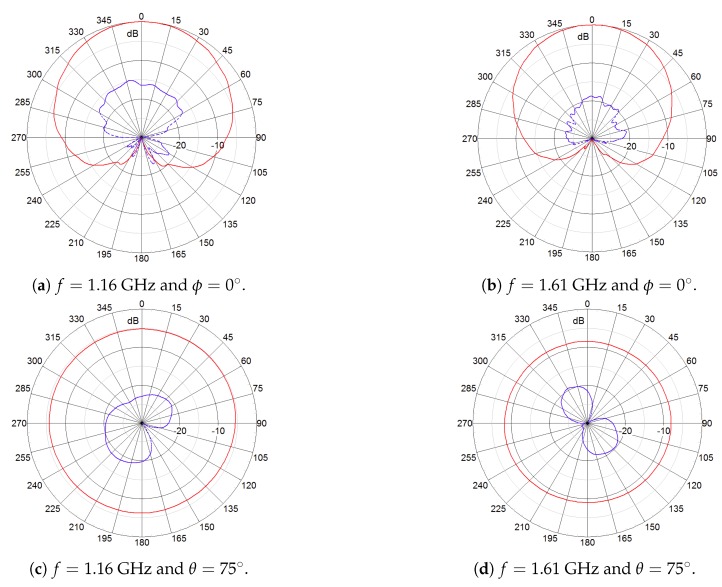
Measured radiation patterns (normalized) of the RHCP output of the 2pol antenna prototype. (Red) RHCP gain and (blue) LHCP gain.

**Figure 3:**
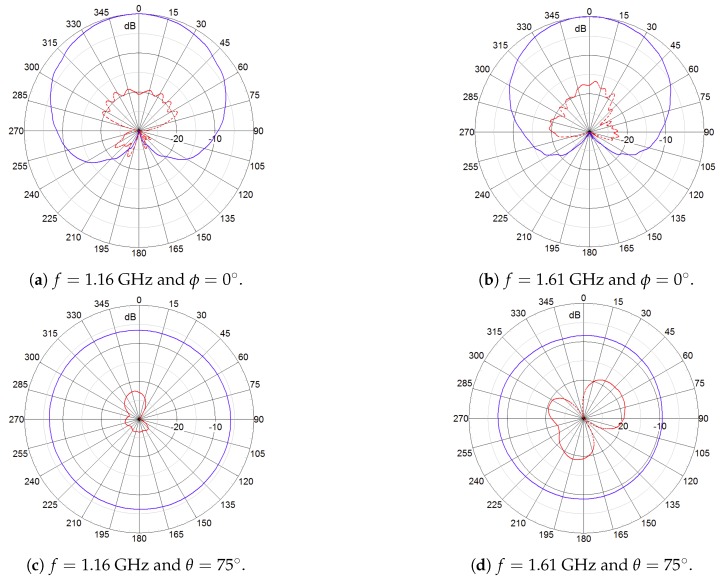
Measured radiation patterns (normalized) of the LHCP output of the 2pol antenna prototype. (Red) RHCP gain and (blue) LHCP gain.

A novel, dual-circularly-polarized (shortly 2pol) antenna has been developed in the project. It relies on a concept that is currently implemented in an RHCP geodetic-grade antenna supporting all GNSS signals in L band [18,19]. Within the framework of the FANTASTIC project, a novel feed network enabling wideband dual-mode operation was developed and, additionally, optimizations in terms of efficiency and beam width were performed. The antenna is illustrated in Figure 1a and its main characteristic are listed in Figure 1b. Figure 2 and Figure 3 show the measured radiation patterns for both the RHCP and LHCP, respectively.

It can be seen that the plots for the co-pol and cross-pol components are almost identical and nearly optimal in terms of multipath suppression and ability to receive signals from satellites at low elevation angles. The antenna provides high polarization purity in all directions of interest with a cross polarization discrimination (XPD: difference between co-pol and cross-pol gain) of at least 10 dB (axial ratio 5.6 dB). This is the minimum value for XPD the antenna will provide, particularly for low elevation angles. For the major part of the specified covering region (higher elevations, directions of interest) the values are greater than 15 dB, being the value in the zenith not worse than 16 dB (axial ratio of 3 dB). It should be noted that a high XPD is very important for the approach described here because of the navigation performance targets, avoiding spill-over of LHCP multipath components into the RHCP line-of-sight signal. It is worth pointing out that the requirement of 16 dB for the XPD is very difficult to achieve at all frequencies and angles of interest. Just for reference, the high-quality antenna Polant MC (not dual circularly polarized) from AeroAntenna, the main competitor due its excellent performance in terms of accuracy, exhibits even worse performance than the FANTASTIC antenna: XPD > 9 dB.

### 2.2. Receiver

The used hardware to capture the data comprised a Septentrio (SSN) AsteRx-U dual antenna multi-frequency receiver in conjunction with the 2pol multi-frequency antenna prototype developed by the Fraunhofer Institute for Integrated Circuits (see Figure 1). The receiver is a commercial L1/L2/E5/E6 receiver used in many industrial positioning and attitude applications. The RHCP output of the antenna was connected to the main input of the receiver, while the LHCP output was connected to the auxiliary input of the receiver. The default software of the AsteRx-U receiver independently acquires and tracks satellite signals from each antenna input. This is a suitable approach for the normal 2D attitude use case of the receiver. However, when used for 2pol applications, the receiver would fail to permanently monitor the polarization of the signal, as the LHCP component could only be tracked if its carrier-to-noise ratio (CN0) is sufficiently high. It is for the above reason that the receiver software was modified.

**Figure 4:**
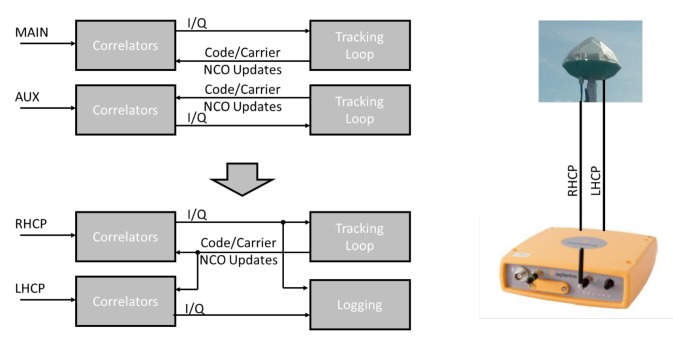
Software modifications included in the AsteRx-U receiver.

Rather than having an independent tracking of the LHCP and RHCP components, the receiver only tracks the RHCP component and replicates the local code and carrier timing of the RHCP tracking to correlators which connect to the LHCP signal, as shown in Figure 4. In this way, the receiver synchronously gathers RHCP and LHCP correlation values, ensuring a permanent polarization monitoring of the signal. Both correlations are integrated coherently over a programmable time ΔT, yielding the following correlation output for satellite signal *k* at epoch *T*:

(1) $$\begin{array}{c} P_{R,k}\left(T\right)≐\int_{T-\Delta T}^{T}\mathrm{RHCP}\left(\tau \right){\mathrm{PRN}}_{k}\left(\tau \right)e^{-j(\omega_{0}\tau +\phi_{k}\left(\tau \right))}{\hat{D}}_{k}\left(\tau \right)d\tau ,\\ P_{L,k}\left(T\right)≐\int_{T-\Delta T}^{T}\mathrm{LHCP}\left(\tau \right){\mathrm{PRN}}_{k}\left(\tau \right)e^{-j(\omega_{0}\tau +\phi_{k}\left(\tau \right))}{\hat{D}}_{k}\left(\tau \right)d\tau . \end{array}$$

The PRN-code and local carrier phase (i.e., $\left\{\tau ,\omega_{0},\phi_{k}\right\}$) are estimated from the RHCP signal, as well as the navigation data bit ${\hat{D}}_{k}$. For pilotized signals, only the pilot component was used in the correlation process, making ${\hat{D}}_{k}$ irrelevant. The integration time ΔT was set to 100 ms. This was done for all satellites in view from GPS (L1C/A-L2C), GALILEO (E1-E5b), GLONASS (L1C/A-L2C/A) and BeiDou (B1-B2). The resulting RHCP and LHCP correlations were logged on non-volatile memory in the receiver for post-processing, along with the usual raw GNSS data and differential corrections from a nearby reference station. At this point, it is interesting to analyze the LHCP SNR after the described processing. The relation between SNR and CN0 is given by SNR=CN0+10log(ΔT). So, for a RHCP CN0 of 40 dB-Hz and RLR around 15 dB, which are typical values under nominal conditions, we get an LHCP SNR of 15 dB for ΔT=100 ms. If we extend the integration time to 1 s, we gain 10 dB, which is sufficient to handle extreme cases with LHCP CN0’s of 10–15 dB-Hz. Lower values are not relevant, as they correspond to RHCP CN0’s useless for precision receiver because of cycle slips.

### 2.3. Data Collection Campaign

**Figure 5:**
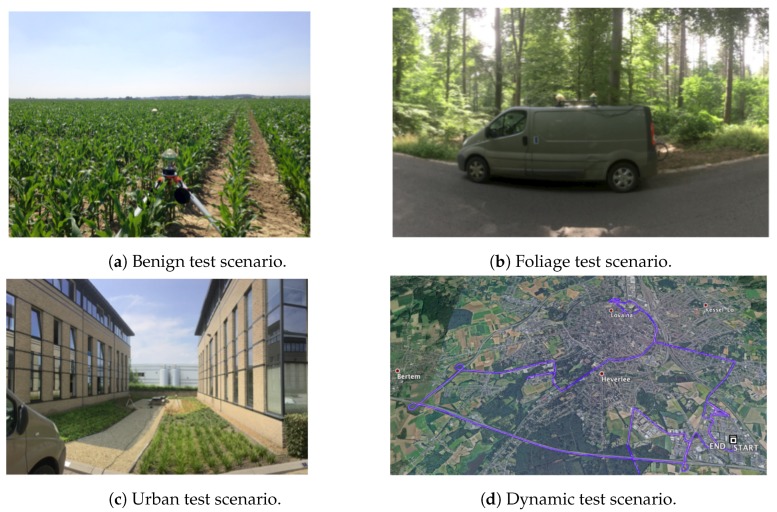
Test scenarios used for the data collection campaign carried out by Septentrio (SSN). (**a**) Static recording attempted to minimize multipath. (**b**) Static recording under trees. (**c**) Static recording between two buildings. (**d**) Truth (pink) and computed (blue) trajectory.

The data collection campaign carried out during the FANTASTIC project was necessary and very useful for the development of the proposed WE function in Section 3. Specifically, the data captured includes the following recordings:

- **Benign**: Static recording at the open sky scenario shown in Figure 5a. The antenna was placed on a tripod on a farmer’s field with dry soil and no objects anywhere nearby. This test is attempted to minimize multipath and to be the reference file for calibration purposes.
- **Foliage**: Static recording under dense tree canopy to capture multipath and/or diffraction, shown in Figure 5b.
- **Urban**: Static recording between two buildings (see Figure 5c). This test is attempted to capture multipath and NLOS conditions.
- **Dynamic**: Dynamic recording with a moving car in a mixed environment including open sky, forests and deep urban scenarios. Figure 5d shows the truth trajectory of the moving car around Leuven, Belgium.

Each recording lasted for about 2 h. The measurements were done during the same time slot at consecutive days. This results in a nearly identical GPS constellation behavior, because of the periodicity of GPS.

**Figure 6:**
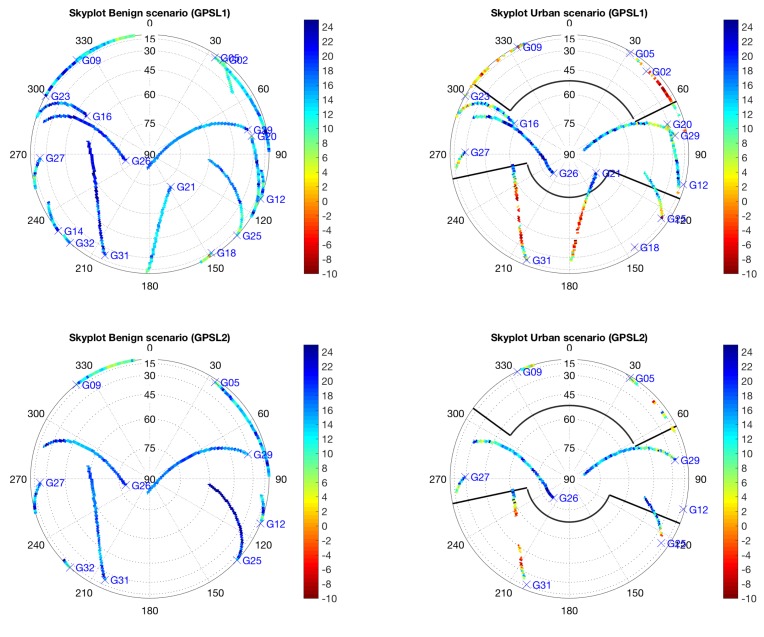

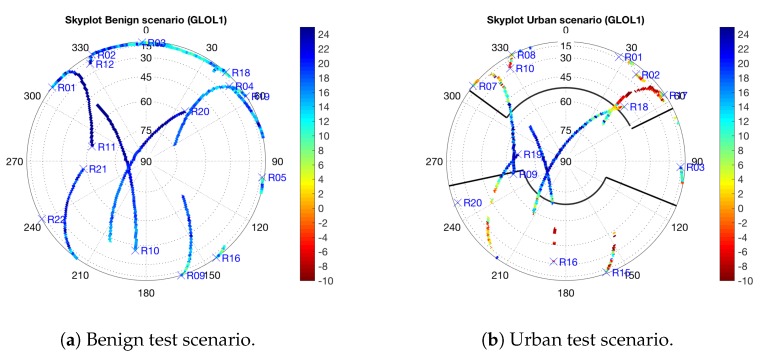
Ratio between RHCP and LHCP components in a sky plot. (**a**) Benign test scenario, and (**b**) Urban test scenario together with the elevation masks corresponding to the buildings in the scenario (see Figure 5a). Results for GPS L1 (up), GPS L2 (middle) and GLO L1 (down).

The goal of these data was to assess the ability of a 2pol antenna system to detect and mitigate multipath. The focus was to evaluate the behavior of the signal strength measured from the LHCP and RHCP components with the elevation angle and multipath environment. To show the capabilities of the 2pol antenna to sense the multipath environment, Figure 6 shows the ratio of the desired RHCP signal component to the spurious LHCP component in a polar azimuth-elevation plot. This was done in the benign environment (see Figure 6a) and the urban environment (see Figure 6b). The antenna has excellent cross-polar ratio in open sky environments and clearly detects signals which have been corrupted by diffraction and reflections. This is indicated with a large positive ratio between RHCP and LHCP components (RLR) (blue color) in the benign scenario and the small and negative RLR (red color) in the harsh environment. We show the results for GPS L1/L2 and GLONASS L1, showing similar results for all the constellations and frequencies. This confirms the good performance of the 2pol antenna designed within the FANTASTIC project, providing similar results for all the frequency bands under analysis. Formally, the RLR is defined as

(2) $${\mathrm{RLR}}_{k}\left(T\right)≐\frac{P_{R,k}\left(T\right)}{P_{L,k}\left(T\right)},$$

with $P_{R,k}\left(T\right)$ and $P_{L,k}\left(T\right)$ the RHCP and LHCP correlation output for satellite *k* at epoch *T*, respectively, given by (1).

## 3. Measurement Weighting and Exclusion (WE)

This section is aimed at explaining the proposed WE function used to reduce the effects of multipath. The development of the WE function is based on a theoretical and experimental assessment of the relation between the signal propagation conditions and the received correlation recordings at both polarizations. For instance, due to signal propagation, if the LHCP component is stronger than the RHCP one, it means that the signal has been received under NLOS conditions [11]. On the other hand, a positive RHCP to LHCP ratio in dBs but with a high signal strength in the LHCP component, would mean that the signal has been received under multipath conditions. In both cases, the user range errors of the corresponding signals would be increased with respect to the nominal errors. In order to mitigate these effects, in the former case it would be interesting to exclude the signal from the PVT computation, whereas in the second case, it would be interesting to weight it down. The key question is how to derive a proper threshold to exclude measurements and a proper function that provides an appropriate value for the weight. In the following, we will provide answers to these two questions. In particular, the rest of the section is organized as follows: Section 3.1 illustrates the design of the exclusion function, whereas Section 3.2 introduces a novel weighting design based on the 2pol antenna.

### 3.1. Measurement Exclusion

**Figure 7:**
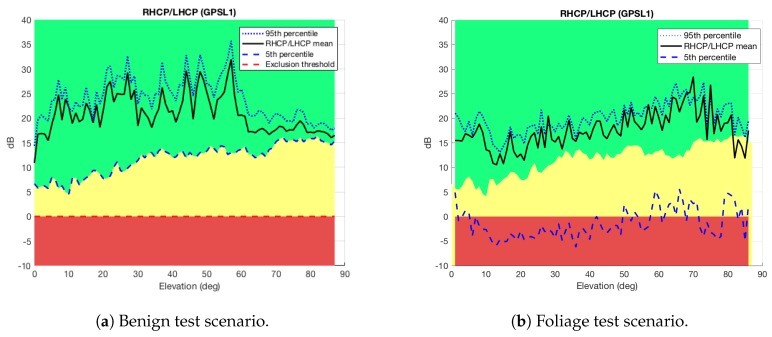
RLR components as a function of the elevation angle for the benign (**a**) and foliage (**b**) scenarios. Three different zones are defined: Severe multipath or NLOS (red), moderate multipath (yellow), and nominal conditions (green).

In order to evaluate the effects of multipath into the 2pol antenna outputs, it is firstly important to analyze the benign scenario (see Figure 5a). This will give us an idea of how the antenna is behaving under nominal conditions. Then, any anomalous behavior departing from the nominal one will be associated to multipath. Specifically, the RLR in the benign scenario is used to determine three different zones as done in Ref. [11] and as shown in Figure 7a, namely

- **Nominal** (green area): Above the fifth percentile of the RLR in the benign scenario. In this area the received multipath is considered to be the one received under nominal conditions and the measurements should be considered by themselves (or traditional weighting).
- **Weighting area** (yellow area): In the range from 0 dB to the fifth percentile of the RLR in the benign scenario. In this area the received multipath is considered to be moderate (subject to a more severe multipath than in the benign scenario) and the measurements should be weighted down.
- **Exclusion area** (red area): Below the exclusion threshold. Theoretically, the exclusion threshold should be a ratio equal to 0 dB. In practice, this threshold must be calibrated. In this area, the received measurements can be considered to be obtained under NLOS conditions, thus they should be excluded.

Furthermore, Figure 7b shows the three different zones together with the mean value of the RLR, as well as the 95- and fifth percentile curves of this ratio, as a function of the elevation angle for the data captured in the foliage scenario. The results clearly suggest the presence of multipath due to the fact that around half of the RLR (see solid black line) measurements lie in the weighting area (yellow area), which is an indicative that these data are contaminated by multipath. Moreover, more than the 5% of the data in the foliage scenario (see fifth percentile line) is in the exclusion area (red area), thus being an indicative of NLOS conditions and very large errors.

These experiments verify the utility of the RLR to identify the presence of multipath on GNSS signals. Now, we have to fix the exclusion threshold used to exclude the measurements. This threshold is of particular interest because it denotes the bound between the cases in which it is useful to mitigate multipath or not. Often, the latter is associated with NLOS propagation for which mitigation has no sense. In Figure 7 this threshold is fixed to 0 dB, which is the ballpark figure for exclusion [11]. Nevertheless, due to the complexity of signal propagation, this threshold may be different. For instance, NLOS can have positive RLR (in dB units) if the reflection incidence angle is above the Brewster’s angle. In addition, LOS may have negative ratio (in dBs) in case multiple LHCP multipath rays interfere constructively.

**Figure 8:**
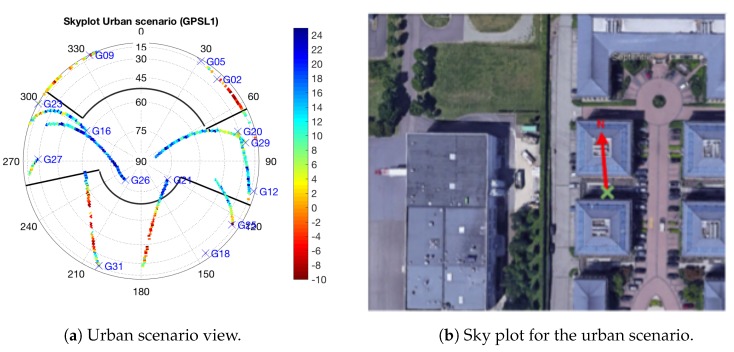
(**a**) RLR in a sky plot for the urban environment. Elevation masks based on the known location of the buildings in the scenario (see (**b**)).

With the aim of fine tuning the exclusion threshold, we will make use of the data collected at the urban scenario. This is so because this scenario includes two buildings in know locations that block the visibility of some satellites. Using the know locations of the buildings we can draw an elevation mask, as shown in Figure 8, in order to know when some satellite is completely blocked by the building, thus if received, it is likely to be received under NLOS conditions. In this way, taking the mean value of the RLR of those satellites obstructed by the buildings we can estimate the value of the exclusion threshold. For instance, we see in Figure 8a that the satellite G31 is obstructed by the southern building from an elevation of 60${}^{\circ }$. Doing so, the selected threshold for the exclusion zone is equal to −1 dB. Results in Figure 7 and Figure 8 are for GPS measurements, but they can be extrapolated for other constellations.

### 3.2. Measurement Weighting

The exclusion of measurements obtained under NLOS conditions is the most appropriate thing to do in current GNSS receivers when using 2pol antennas. Nevertheless, when the LOS is present we can do better trying to reduce the effects of multipath into the obtained measure. To do so, three different concepts were proposed in Ref. [9], namely

1. **Measurement weighting**: The effects of multipath into the PVT solution can be reduced by estimating the standard deviation of the range measurements due to multipath and pass it to the navigation processor, so that the measures can be weighted accordingly [10,20].
2. **Range correction**: The idea is to estimate the offset included by multipath into the range measurements and apply them directly into the provided measurements [21].
3. **Tracking correction**: The idea here is to use a common tracking loop for both RHCP and LHCP components that is closed using together the outputs of both the RHCP and LHCP tracking outputs to mitigate multipath [6].

So, whenever NLOS signals are detected (exclusion area) these should be discarded from the navigation solution. The rest of signals should be used after applying some multipath countermeasure.

In this paper, we will focus on the measurement weighting solution. Specifically, in order to estimate the standard deviation of the measurements we will use an experimental model based on the RHCP and LHCP components, namely

(3) $$\sigma =f\left(P_{L},P_{R},\mathrm{CN}0_{R};a,b\right),$$

where $P_{L}$ and $P_{R}$ stand for the prompt correlator value of the LHCP and RHCP component, respectively, $\mathrm{CN}0_{R}$ the CN0 of the RHCP component, and {a,b} are the model parameters. The form of the model (i.e., f(·)) may be derived theoretically, but the parameters have to be determined empirically. In doing so, we will take into account several practical aspects such as receiver design and/or antenna artifacts. A proper model may be of the form

(4) $$f\left(P_{L},P_{R},\mathrm{CN}0_{R}\right)=\sigma_{0}\left(\mathrm{CN}0_{R}\right)\left[1+c_{1}\frac{P_{L}}{P_{R}}\right]+c_{2},$$

where $\left\{c_{1},c_{2}\right\}$ are two constants, and $\sigma_{0}\left(\cdot \right)$ the traditional model for the standard deviation used for CN0- or elevation-based weighting [20,22]. The weighting function parameters are optimized by using PVT residuals statistics.

The idea in Equation (4) is to add a correction factor to the traditional weighting models. This correction factor increases as long as the LHCP to RHCP ratio increases (i.e., the RLR decreases), thus increasing the modeled standard deviation as long as the multipath effects are stronger (with respect to the LOS signal). This concept is similar as the one adopted in Refs. [23,24] in order to model NLOS and ionospheric errors, respectively. Specifically, we consider the following model:

(5) $$\sigma^{2}=a\frac{1}{\mathrm{CN}0_{R}}\left[1+c\frac{P_{L}}{P_{R}}\right]=a\frac{1}{\mathrm{CN}0_{R}}+b\frac{P_{L}}{\mathrm{CN}0_{R}P_{R}},$$

with σ the standard deviation of the measurement error. From Equation (4), we have used $c_{1}=c$ and $c_{2}=0$ in Equation (5) and the traditional CN0 weighting model.

**Figure 9:**
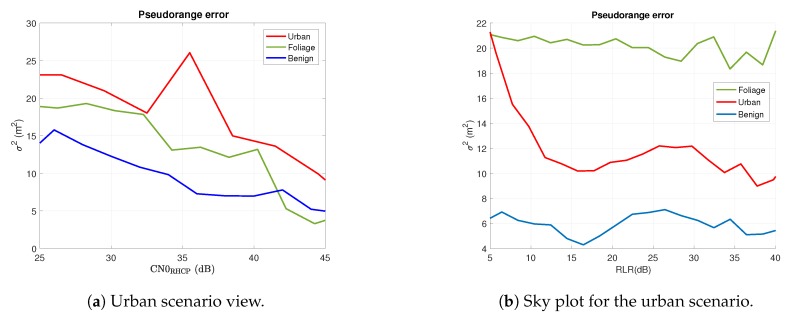
Comparison of the pseudorange error as a function of the CN0 (**a**) and RLR (**b**) for different scenarios. The gap between curves is due to multipath effects to be modeled through the LHCP component.

**Figure 10:**
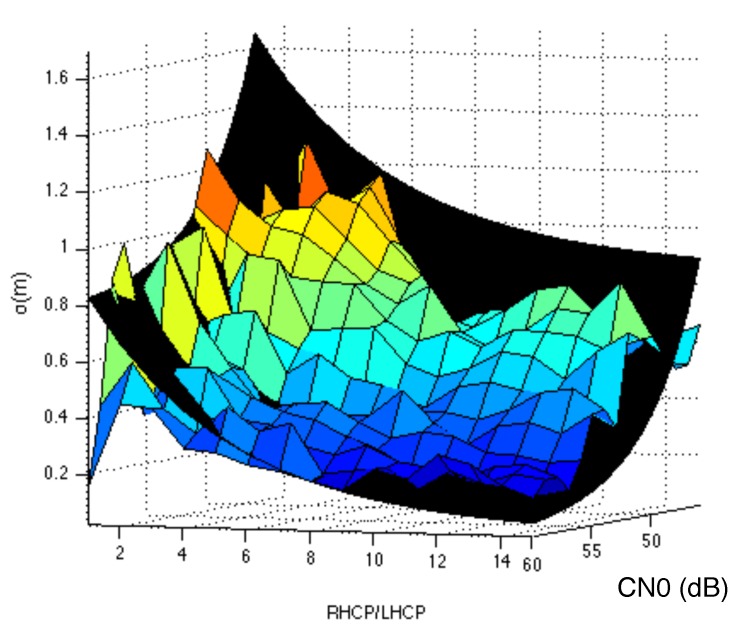
Least-Squares 3D fitting of the measured phase errors as a function of the RHCP to LHCP ratio and the CN0 of the RHCP component. Empirical data (colored) and fitted shape (black).

As already stated, the model parameters {a,b} should be determined empirically. Specifically, in order to include a proper correction factor, a two-dimensional optimization will be considered. This is for making sure that the correction factor included in Equation (5) increases when the multipath effects are stronger and not because any other effect such as signal attenuation. This fact is illustrated in Figure 9 in which we see how for a given value of CN0 in the RHCP component we have different pseudorange errors depending on the scenario we are. This difference of error for a given RHCP CN0 value is mainly due to the multipath effects (scenario dependent), which will be visible in the LHCP component. For this reason, a two-dimensional or equivalently a 3D-shape fitting will be performed. This is illustrated in Figure 10 in which the shaded-shape is the resulting fitting after estimating the model parameters in Equation (5) using the empirical data shown as the colored shape. It should be noted that the fitting is performed using all the collected data (i.e., including all scenarios). This increases the number of samples used to estimate the error for a given value of CN0 and RLR. So, we have smooth transitions avoiding abrupt peaks in the experimental data such as the one experienced in Figure 9 for the Urban curve at CN0 = 36 dB-Hz.

**Figure 11:**
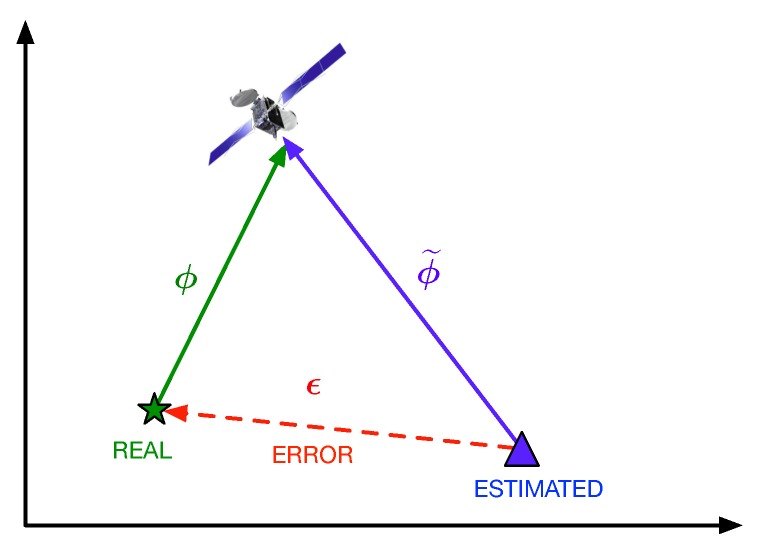
Illustration of the phase error computation based on the positioning error.

Before estimating the model parameters, let us first talk about the empirical computation of the range errors. Indeed, both pseudorange and phase measurements should be analyzed. In order to empirically compute the measurement errors, for the pseudorange, we use the traditional code-minus-carrier (CMC) iono-free combination. For the phase error, let us define the residual phase error, $\epsilon_{r}$, as the difference between the measured phase, $\phi_{m}$, and the one obtained from the estimated PVT solution, $\tilde{\phi }$. Then, we have

(6) $$\epsilon_{r}≐\phi_{m}-\tilde{\phi }=\phi_{m}-\tilde{\phi }+\phi -\phi =\epsilon_{\phi }+\phi -\tilde{\phi },$$

with ϕ the real phase and $\epsilon_{\phi }≐\phi_{m}-\phi$ the phase measurement error. It is worth pointing out that the term $\phi -\tilde{\phi }$ is equivalent to the projection of the error vector, ϵ, into the truth range vector, $\hat{\rho }$ (see Figure 11). For clarification, we have the tuple $\left\{\phi ,\phi_{m},\tilde{\phi }\right\}$ corresponding to the real (unknown) phase, the measured phase provided by the GNSS receiver, and the phase we would get from the estimated PVT to the given satellite, respectively. It is worth noting that $\tilde{\phi }$ is used here in order to get the following formula for the phase error, which can be easily computed with the available data:

(7) $$\epsilon_{\phi }=\epsilon_{r}-\epsilon^{⊤}\hat{\rho },$$

where ϵ is the 3D vector given by the difference between the estimated PVT and the real position, and $\hat{\rho }$ is the unitary vector of the direction between the real position and the satellite. Summarizing, to compute the phase error, we use the phase residual error minus the projection of the PVT error into the truth range, which is available data within the FANTASTIC project.

Once both pseudorange and phase measurement errors are calculated, the next step is to estimate the model parameters from this data. For the sake of notation simplicity, let us define $x_{i}≐\mathrm{CN}0_{R}\left(i\right)$ and $y_{i}\;≐\;P_{L}\left(i\right)/P_{R}\left(i\right)$ with i=1,⋯,N the measurement index. Let also $\sigma^{2}\left(i\right)$ be the measured root mean square (RMS) error of the range measurements. Therefore, from Equation (5), we can write

(8) $$\sigma^{2}=\left[\begin{array}{c} \sigma^{2}\left(1\right)\\ \sigma^{2}\left(2\right)\\ \vdots \\ \sigma^{2}\left(N\right) \end{array}\right]=\left[\begin{array}{cc} \frac{1}{x_{1}} & \frac{y_{1}}{x_{1}}\\ \frac{1}{x_{2}} & \frac{y_{2}}{x_{2}}\\ \vdots & \vdots \\ \frac{1}{x_{N}} & \frac{y_{N}}{x_{N}} \end{array}\right]\cdot \left[\begin{array}{c} a\\ b \end{array}\right]=H\theta ,$$

and the model parameters can thus be estimated by a least-square fitting as

(9) $$\hat{\theta }=\left[\begin{array}{c} \hat{a}\\ \hat{b} \end{array}\right]={\left(H^{⊤}H\right)}^{-1}H^{⊤}\sigma^{2}.$$

Then, the standard deviation to feed the PVT engine at time *n* can be estimated as a function of $x_{n}$ and $y_{n}$ by

(10) $$\hat{\sigma }\left(n\right)=\sqrt{\hat{a}\frac{1}{x_{n}}\left(1+\frac{\hat{b}}{\hat{a}}\frac{y_{n}}{x_{n}}\right)}.$$

## 4. PVT Results

In the previous sections we have demonstrated the capability of the 2pol antenna to detect the presence of NLOS (see Figure 8) and to provide a good match between the proposed measurement weighting and the measured error standard deviations (see Figure 10). Now, it is time to see the effects of exploiting these capabilities on the performance of the PVT solution. Nevertheless, first we have to fine-tune some parameters related to the developed WE function. In this section, we analyze the PVT results for several configurations in order to fine-tune these parameters. To do so, an iterative process, combining commercial tools and new software developed within the framework of the FANTASTIC project, has to be applied. In particular, Section 4.1 explains the process carried out to obtain the results analyzed in this section. Section 4.2 shows the fine-tuning of the proposed algorithms in real-world conditions (i.e., using the collected data), and then Section 4.3 gives a summary of the obtained PVT results.

### 4.1. Processing of the Collected Data

**Figure 12:**
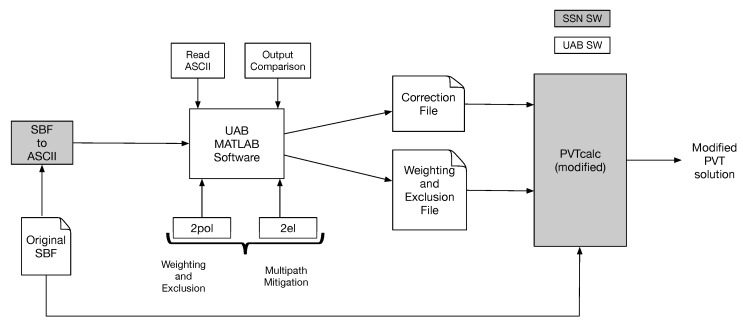
Sketch of the procedure carried out to obtain the results presented in Section 4.

In order to apply the developed WE function to the data captured in the project and evaluating its effects into the PVT solution, we use a set of software (SW) commercial tools provided by SSN and developed by Universitat Autònoma de Barcelona (UAB). A sketch of the procedure carried out to obtain the results presented in this section is shown in Figure 12, in which the grey blocks correspond to SW provided by SSN and the white blocks are those developed by UAB. As shown, the first step is to convert the original SBF files provided by SSN to ASCII files, so that the data can be read and processed by MATLAB. Thanks to the modifications of the PVTcalc SW provided by SSN, the outputs of the UAB software must be two different files including corrections and exclusion flags to be applied in the PVT computation. These files are inputs to the PVTcalc SW, which carries out all the needed corrections and modifications to apply the developed algorithms by UAB into the PVT computation. The PVTcalc SW is the proprietary SSN positioning engine and it was derived from the post processing engine of the commercial PP-SDK package [25].

In this paper, we focus on the WE function developed in the FANTASTIC project, so the presented results in this section will be based only on this part of the project. The results about multipath mitigation is out of the scope of this paper. So, the results presented here, only use the WE flags; that is, depending on the RLR of the 2pol antenna, we will decide whether to exclude the measurement or to apply weighting. This will be decided based on the discrimination between LOS and NLOS conditions. Finally, after applying the developed WE function we have to evaluate the resulting PVT solution. With this aim we have generated a script to compare two different output files. The idea is to compare PVT results such as the RMS 3D error (calculated with a truth reference); percentage of time of each PVT mode, RMS errors for each PVT mode, etc.

Before entering into the detailed analysis of the PVT results, let us introduce the general structure and terminology used when analyzing the collected data. The stored I/Q correlation data at each scenario is used to generate the correction and weighting and exclusion files to be fed to the PVTcalc (see Figure 12), thus generating the PVT solution after applying the algorithms. This PVT solution is computed using all the available constellations (i.e., GPS. GLONASS, GALILEO and BeiDou), and then it is compared with the PVT solution obtained without applying the algorithms; that is, the solution using the one antenna configuration. This solution will be referred to as the reference file. Furthermore, the traditional measurements of the GNSS receiver are useful to extract the error statistics of the PVT solutions. For this task the real position of the receiver is of particular importance. For the static scenarios, this position is obtained by averaging the PVT solution among the whole file. Only the RTK fixes are averaged, thus providing a very accurate estimate of the real position. For the dynamic scenario, the real position of the receiver at each time was know thanks to the use of a high-end GPS Inertial Navigation System (INS). This system provides a very accurate position useful to evaluate the positioning error of the gathered data in this scenario. Particularly, the used INS was the commercial product named ATLANS-C of ixblue with specifications given in Ref. [26].

With the real position of the receiver we are able to compare the error statistics of the reference (i.e., one antenna configuration) and the 2pol-enhanced PVT solution. Specifically, one of these statistics is the total 3D RMS error. This error is computed as the RMS error between the real position (computed as before) and the analyzed PVT solution. Moreover, we are focused on the performance of the RTK positioning mode. This is why we will also consider the RMS error but only taking into account those epochs with a particular PVT mode. Specifically, we will consider the RTK fixed and RTK float modes, which are phase-based positioning modes (with fixed or float ambiguities, respectively). Finally, we will also analyze the same results but for the RTK fixed mode when excluding wrong fixes. The wrong fixes are defined as those fixes showing an error greater or equal than 10 cm.

### 4.2. Fine-Tuning of the WE Function

Next, we show the fine-tuning of the developed WE function. To do so, we show the results obtained with different exclusion thresholds and several averaging configurations. Each averaging configuration accounts for a different averaging time in seconds, meaning that we are averaging the RLR values over the averaging time. We do so for all the considered scenarios. The aim is to find the best configuration in terms of PVT performance. At this point, it is worth highlighting that the results shown in this section only used the exclusion function. This is because the application of the weighting function did not provide a valuable effect into the PVT performance. This will be further analyzed in Section 4.3.

#### 4.2.1. Static Urban Scenario

**Figure 13:**
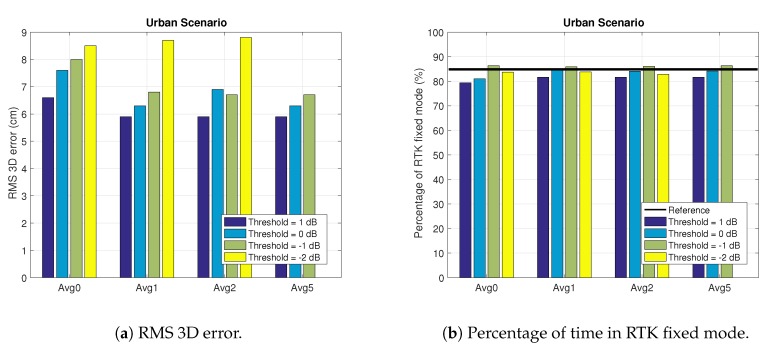
Results in the Urban scenario for different exclusion thresholds and average configurations. The reference value is omitted in (**a**) due to the large difference with the values illustrated in the plot.

Let us analyze the static urban scenario. The results of analyzing different exclusion thresholds and average configurations are shown in Figure 13 and Table 1. We see that for all the thresholds the results are better when we average the RLR values. Particularly, the best results (in terms of percentage of RTK fixes) are obtained when applying the exclusion threshold of −1 dB. Regarding the averaging configuration, for the case of a threshold of −1 dB, the best configuration is the one with an averaging time of 5 s. Nevertheless, for the rest of analyzed thresholds the best averaging configuration is obtained with an averaging time of 1 s. It is interesting to note that the analyzed [−2,1] range of thresholds is representative to find the best exclusion threshold. The reason is that larger thresholds reduce the RMS error but at the expense of reducing the percentage of RTK fixes. The reason is that the larger the thresholds the larger the number of excluded satellites. On the other hand, lower thresholds would produce worse results. The reason is that the number of excluded satellites is reduced, then the results would be more similar to the reference ones. Similar results are obtained for the rest of scenarios, so we will show results for thresholds within the [−2,1].

**Figure 14:**
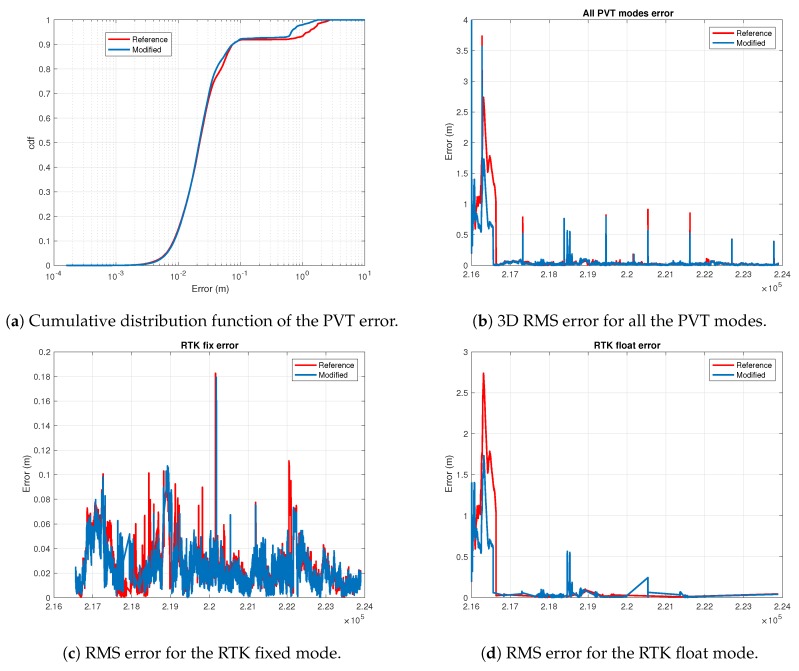
Graphical results of the PVT error when applying (blue curves) or not (red curves) 2pol exclusion in the urban environment.

It is worth pointing out that we focus on improving the performance of the RTK phase-based positioning mode. This is why we consider the percentage of RTK fixes as the parameter to optimize. This is illustrated in Figure 13a, which shows how the best configuration in terms of 3D RMS error is the one with threshold of 1 dB and averaging time of 1 s. Notwithstanding, Figure 13b shows how the best configuration in terms of RTK fixes is the one using an exclusion threshold of −1 dB. Therefore, we say that the last one is the best configuration for the Urban scenario. Actually, we see in Figure 13b that the exclusion threshold of −1 dB is the only one that gives an improvement with respect to the reference one (i.e., results without applying the exclusion function). Last but not least, we see an important improvement of the error in the RTK float mode, being greater than a 50% reduction for all configurations. The reason of this improvement is the exclusion of NLOS ranges, which were tremendously affecting the range accuracy. Moreover, there is a conversion effect from RTK fixes into RTK float, thus improving the average accuracy of the RTK float mode. Similar conclusions can be extracted from the results shown in Figure 14. When analyzing the cumulative distribution function (cdf) of the PVT error we do not see a valuable improvement between the solution when applying (blue curve) or not (red curve) 2pol exclusion. This improvement is apparent when comparing the total 3D RMS error as shown in Figure 14a. Indeed, as shown in the plots of the lower row of Figure 14, the improvement on the total error comes from the improvement of the RTK float error, the RTK fix error is very similar when applying or not 2pol exclusion.

#### 4.2.2. Foliage Scenario

**Figure 15:**
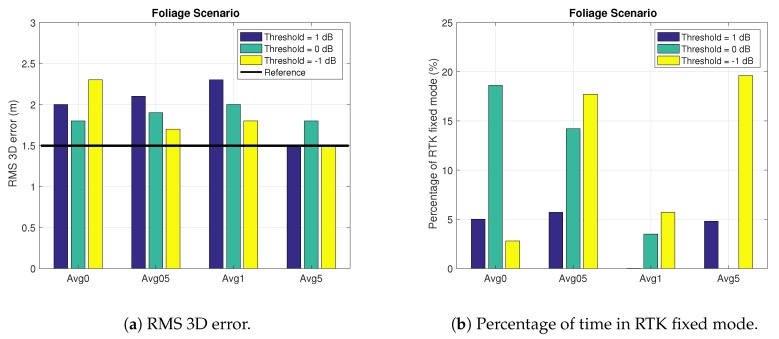
Results in the Foliage scenario for different exclusion thresholds and average configurations. The reference value is omitted in (**b**) due to the large difference with the values illustrated in the plot.

Let us analyze the Foliage scenario for which we have the results shown in Figure 15 and Table 2. In this case, the configuration with an exclusion threshold of −1 dB also provides the best results. The best averaging configuration, though, is the one with an averaging time of 5 s. These results are corroborated with Figure 15b, in which we see that the best results in terms of RTK fixed mode are given with −1 dB threshold, for any averaging time greater than 0 s. Notwithstanding, we see in Table 2 that the percentage of corrected RTK fixes mode for the reference file (i.e., 29.57%) is greater than for any other configuration.

One interesting thing for this scenario can be observed in Figure 15a. We see that the configuration with an exclusion threshold of −1 dB, for an averaging time greater than 0 s, also gives the best results in terms of RMS 3D error. This error even gets smaller than the reference one for the configuration with an averaging time of 5 s. In this scenario, the RTK float error is also reduced but not in the same percentage as in the urban scenario. The reason is that in the foliage scenario the reduction in percentage of RTK fixes is not directly translated into an increment of the percentage of RTK float. That is, from the 10% reduction of RTK fixes, only the 4% is translated into float mode; the other 6% moves to code-based positioning.

The reason for the improvement on the RTK float mode is the same as for the urban scenario. The reduction of the RTK fixes here is due to the fact that when excluding satellites we loose redundancy to fix the phase ambiguities, thus reducing the percentage of RTK fixes. Moreover, in the foliage scenario, the remaining satellites after the exclusion are still affected by multipath, thus worsening the performance to fix the phase ambiguities.

#### 4.2.3. Dynamic Scenario

**Figure 16:**
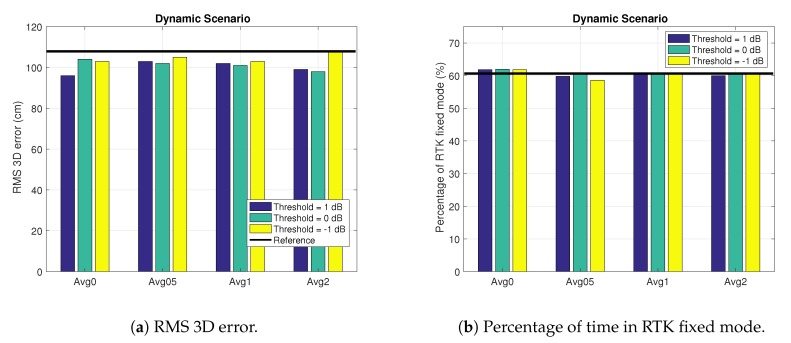
Results in the Dynamic scenario for different exclusion thresholds and average configurations.

Finally, we show the results for the dynamic scenario in Figure 16. We see a reduction of the 3D RMS error for all configurations in Figure 16a. Moreover, we also see that the best results are obtained without averaging (i.e., Avg0) and with an exclusion threshold of 1 dB. On the other hand, in Figure 16b we see that the best configuration in terms of percentage of RTK fixes is given by the configuration with a threshold of 0 dB (and Avg0). Based on these results and additional experiments carried out during the project, we choose the configuration with threshold of 1 dB without averaging as the best configuration both in terms of 3D RMS error and % of RTK fixes. Here, in the dynamic scenario, it is worth pointing out that the percentage of RTK fixes for the configuration without averaging is improving the value given by the reference file. So, in the dynamic scenario we obtain better results than for the foliage scenario, but they are worse than the ones obtained in the urban scenario.

### 4.3. PVT Results Summary

After showing and analyzing the fine-tuning of the exclusion function for all the considered scenarios, it is time to summarize the best results obtained in each scenario and compare them. Table 3 provides the most important results to evaluate the effects of the developed WE function into the PVT performance. In particular, the second column of the table shows the relative improvement (in terms of RMS 3D error) of using the 2pol antenna, with respect to the regular configuration (i.e., using 1 antenna). We see how the positioning performance in terms of RMS 3D error is improved with the use of the 2pol antenna. This improvement is not big in the foliage and dynamic scenarios, a reduction of 3% and 11% of the 3D error, respectively. Nevertheless, this is not the case in the static urban scenario, in which the 2pol antenna provides a reduction of almost 50% of the original error. The reason of these results can be explained by the type of NLOS propagation in each scenario. It is known that a long delay (distant reflector) introduce a large position error, whereas a short path delay (near reflector) have a much smaller effect. It is likely that the NLOS propagation in the static urban scenario comes from distant reflectors, thus causing large errors. This fact would explain the large improvement in the urban scenario, with respect to the foliage scenario, in which the reflections might come from near reflectors.

It is worth pointing out that the presented results were obtained using RTK phase-based positioning, thus providing high-accuracy performance around 5 cm to 2 m for the fixed and float RTK mode, respectively. The percentage of time that the receiver is able to fix the phase ambiguities is shown in the third column of Table 3. The percentage of time in RTK fixed mode is particularly high in the urban scenario, being greater than 80%, and dynamic scenario, which is more than the 50%. The rest of time the receiver is operating in RTK float mode, except in the dynamic scenario in which a 20% of the time the receiver is in code-based positioning mode. This distribution of errors and percentage of time is the reason of the difference of the improvement in terms of RMS error between scenarios. Another explanation of these results is the fact that the accuracy of the position solution obtained after excluding signals depends on the quality of the remaining signals and the quality of the user-satellites geometry. For instance, results in Table 3 show how the application of the exclusion function do not improve the percentage of time that the receiver is working with RTK fixed mode, being even worse in the foliage scenario. The reason is that in this scenario, most signals were contaminated by multipath, NLOS reception and/or diffraction. In such a case, when excluding signals, the quality of the remaining signals might be worse than the excluded ones, being thus more difficult for the receiver to fix the phase ambiguities. As a result, in the foliage scenario, those epochs that the receiver is not able to fix the ambiguities become in float mode. On the contrary, in the urban scenario, there were several signals with good visibility and few signals with severe multipath (see Figure 8). Thus, when excluding the severe multipath we improve the accuracy because we have good signals with good geometry. Nevertheless, this accuracy is not reflected in an improvement of the resolution of phase ambiguities, which is maintained, but in the reduction of the RTK float mode error. Similar arguments hold for the dynamic scenario.

**Figure 17:**
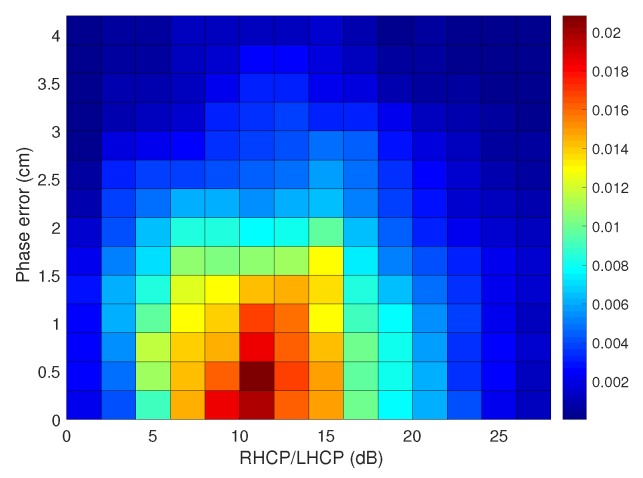
Relative frequency of the phase error measurements as a function of the RHCP to LHCP ratio.

So, in general, the improvements on the positioning accuracy shown in Table 3 come from the improvements on the RTK float mode error. The RMS 3D errors for the RTK float mode for all the analyzed scenarios are collected in the fourth column of Table 3. Finally, it is worth noting that the results that we have presented in this section were obtained only applying the exclusion function. This is because the application of the weighting function did not provide a valuable effect into the PVT solution. The reason is explained with the results in Figure 17, which shows the relative frequency of the phase error as a function of the RLR. That is, the percentage of measurements that lies in a given grid of error for a given value of RLR. We see in the figure that we have small errors even for small values of the RLR, and viceversa. With this kind of behavior is difficult that the used weighting function provides a proper fit useful to improve the positioning performance.

## 5. Conclusions

A novel weighting and exclusion function has been proposed and demonstrated using real data collected within the framework of the FANTASTIC project. The technique uses the RHCP and LHCP components of a 2pol antenna. Depending on the difference between the strength of these two components, the technique either excludes or weighs the measurements. We have demonstrated the detection of NLOS and the improvement of removing these signals from the PVT computation. For the first time, we have assessed the effects of this exclusion into the RTK phase-based positioning. In general, we can conclude that exclusion is beneficial for RTK positioning because the RMS 3D error is reduced. It is worth noting, though, that this improvement is dependent on the scenario. Actually, the improvement depends on the NLOS propagation (i.e., small or large delay) and the quality of the remaining signals and user-satellite geometry. It is for the above reason that, as indicated by the obtained results, the exclusion function is very useful in urban environments or those environments with large delay NLOS propagation and/or scenarios in which most signals have good visibility and there are few signals with severe multipath. So, the improvements gained with the exclusion function come from the exclusion of NLOS measures, this is something that cannot be achieved with traditional mitigation techniques [1,2], therefore the large improvements experienced in the urban scenario.

As already stated, the results presented in this paper, show the application of the concept of 2pol exclusion into RTK positioning. This is in contrast to [12], which applies a weighting function with a 2pol antenna for precise point positioning. We have also proposed a weighting function with a good fitting between the used model and the measured data. Notwithstanding, this fitting was not translated into a valuable improvement of the PVT solution. The reason is that in RTK phased-based positioning the phase measurements used for the PVT computation are accurate measurements with similar errors (of the order of few cm). Then, the weights provided by the weighting function will be similar, thus the effects on the PVT solution will be negligible. In summary, we have proposed a novel technique to mitigate the effects of multipath into the PVT solution. The technique can be easily implemented in any receiver with a two-antenna input feed, allowing the use of a 2pol antenna. This is in contrast to traditional techniques, which are usually too complex to be implemented in practice. Moreover, the proposed technique is useful for dynamic scenarios without needing any additional component such as maps or cameras. This is an advantage with respect to those contributions proposing site-dependent techniques. Finally, regarding multi-antenna solutions (best candidates nowadays), our contribution provides an advantage in terms of dimensions. So, the simplicity, robustness, and size of the proposed solution is expected to leverage the application of precise GNSS positioning in many professional applications in which current GNSS accuracy is insufficient.

## Figures and Tables

**Table 1:** Static urban scenario: PVT results for different exclusion thresholds and average configurations.

Configuration	Total	RTK_Fixes		RTK Correct Fixes (Wrong Fix: Error > 10 cm)		RTK_Float	
	Error (m)	Error (cm)	%	Error (cm)	%	Error (cm)	%
Reference	14.0	2.5	85.2	2.5	85.1	79.9	14.7
Threshold = 0 dB							
Avg1	6.7	2.4	84.7	2.4	84.7	26.7	15.3
Threshold = −1 dB							
Avg5	6.7	2.6	86.3	2.6	86.3	31.6	13.7

**Table 2:** Foliage scenario: PVT results for the best configuration in terms of exclusion threshold and average configuration (i.e., Avg5 and Threshold = −1).

Configuration	Total	RTK_Fixes		RTK Correct Fixes (Wrong Fix: Error > 10 cm)		RTK_Float	
	Error (m)	Error (cm)	%	Error (cm)	%	Error (m)	%
Reference	1.5	5.9	32.8	5.0	29.6	2.1	63.7
Avg5 + Th =−1	1.5	18.9	27.9	5.1	19.6	1.9	67.3

**Table 3:** Comparison of the results of some parameters for the three considered scenarios: Relative improvement in terms of RMS 3D error, the percentage of time in RTK fixed mode and RMS 3D error in the RTK float mode when applying exclusion or not.

Scenario	Relative Improvement RMS 3D Error	RTK Fixed (% of Time)		RTK Float RMS 3D Error (cm)	
		One Antenna	2pol Exclusion	One Antenna	2pol Exclusion
Static Urban	50%	85	85	80	37
Static Foliage	3%	29	19	213	186
Dynamic	11%	61	62	96	88

## Abbreviations

2pol	dual-circularly-polarized
CMC	Code-Minus-Carrier
CN0	carrier-to-noise ratio
GNSS	Global Navigation Satellite System
GSA	European GNSS Agency
IMU	Inertial Measurement Unit
INS	Inertial Navigation System
LHCP	Left-Hand Circular Polarization
LOS	Line-of-Sight
NLOS	Non-Line-of-Sight
PVT	Position, Velocity and Time
RHCP	Right-Hand Circular Polarization
RLR	RHCP to LHCP Ratio
RMS	Root Mean Square
RTK	Real-Time Kinematics
SSN	Septentrio
SW	SoftWare
UAB	Universitat Autònoma de Barcelona
WE	Weighting and Exclusion
